# Analysis of BnMTL, a novel metallothionein‐like protein in the bast fiber crop Ramie (*Boehmeria nivea*)

**DOI:** 10.1002/2211-5463.12705

**Published:** 2019-08-18

**Authors:** Gang Gao, Jikang Chen, Ping Chen, Kunmei Chen, Aminu Shehu Abubakar, Chunming Yu, Aiguo Zhu

**Affiliations:** ^1^ Institute of Bast Fiber Crops Chinese Academy of Agricultural Sciences Changsha China

**Keywords:** BnMTL, Cd^2+^ tolerance, expression pattern, heterologous expression, ramie

## Abstract

Ramie (*Boehmeria nivea*) is a perennial herb that is highly tolerant of heavy metals. In the present study, we cloned a novel metallothionein‐like gene from ramie; this gene, termed *BnMTL*, encodes a putative 46 amino acid protein with a molecular mass of 4.38 kDa. Analysis using quantitative RT‐PCR revealed that cadmium (Cd^2+^) treatment results in elevated expression of *BnMTL* in the roots. We heterologously overexpressed *BnMTL* in *Escherichia coli* cells to examine its binding to Cd^2+^ and its possible role in homeostasis. Recombinant *E. coli* cells expressing *BnMTL* exhibited a high tolerance of Cd^2+^ stress up to a concentration of 1 mm, and the observed accumulation of Cd^2+^ was almost eight‐fold higher than the control. These results demonstrate that *BnMTL* (i) is highly expressed in the root following exposure to Cd^2+^ and (ii) encodes a typical metallothionein‐like protein with high cadmium‐binding activity.

**Abbreviations**
IPTG: isopropyl‐d‐thiogalactoside
qRT‐PCR: quantitative RT‐PCR

Plants have developed a suitable mechanism for controlling and responding to the uptake and accumulation of heavy metals that are considered as critical environmental contaminants of great concern with respect to the ecological environment and also pose nutritional and evolutionary threats 1. Cadmium (Cd^2+^) is one of such heavy metal, although it occurs in trace quantities, yet causes toxic effects to both plants and animals 2, 3, 4. Growing interest in molecular genetics and transgenic plants has increased our understanding of mechanisms of heavy metal tolerance in plants and opens new possibilities with respect to phytoremediation.

Phytoremediation as a perspective technology of soil clean‐up has been intensively studied as a result of its low cost, environmental aesthetics and *in situ* effective treatment. The phytoremediation technique for treating heavy metal contaminated soil includes phytoextraction, phytostabilization, rhizofiltration and phytovolatilization 5, 6. Recent progress in phytoremediation aiming to curb heavy metal pollution has focused on the screening of suitable plant species that are potential heavy metal accumulators, such as *Solanum nigrum* 7, *Helianthus annuus* 8, *Sorghum bicolor* 9, *Zea mays* 10 and *Boehmeria nivea* 11. Ramie (*Boehmeria nivea*) is conisidered to have excellent qualities for enduring heavy metal pollution and is considered as an ideal economic crop for the phytoremediation of mild or moderately heavy metal polluted areas as a result of several ramie varieties performing hyperaccumulative characteristics on heavy metals 12.

Ramie is a perennial fiber crop with high biomass and strong root system. Moreover, the ramie fiber is mainly produced as textile raw material, and this may help minimize the potential hazards of bringing toxic metals into the food chain. Some wild genotypes were dominant in the smelter tailings, highlighting their hyperaccumulation properties. Ramie genome 13 and genome‐wide expression profiles 11 will aid the identification, quantification and annotation of key genes related to heavy metal tolerance. This will also be helpful for target discovery and pathway studies.

Metallothioneins are small proteins that appear to play key role in heavy metal homoeostasis 14. Several plant metallothioneins have been overexpressed and heterologously expressed in microbial hosts aiming to examine the metal binding properties of these proteins and their ability to exert heavy metal tolerance 15, 16, 17. Such studies have provided important evidence indicating that plant metallothioneins are capable of providing a biological function and a metal tolerance ability in nonplant systems. Plant metallothioneins are cysteine‐rich polypeptides with a cysteine content varying between 10 and 17 residues. The large number of cysteine residues binds a variety of metals by mercaptide bonds. Although many metallothionein and metallothionein‐like proteins have been identified in plants 18, 19, there are still some difficulties with respect to the functional characterization of these proteins because of the instability of metallothionein in the presence of oxygen 20.

The present study therefore aimed to (i) clone and identify the putative metallothionein‐like protein encoding gene; (ii) determine its regulation under Cd^2+^ stress in the ramie; and (iii) assay its expression patterns at various Cd^2+^ concentration levels. Its Cd^2+^ binding properties and possible roles in detoxification were also evaluated by heterologous expression in *E. coli* cells.

## Materials and methods

### Plant growth and Cd^2+^ stress treatment

Ramie plants were cultured in hydroponic system, as described previously 21. Non‐lignified tender shoots (12–15 cm long) with two or three leaves were cut and soaked in 0.1% carbendazim for 5 min. The sterilized shoots were then transferred to a hydroponic apparatus, with distilled water being used as the solution to induce aquatic root germination, which was later replaced with nutrient solution. The set‐up was placed in greenhouse under a 14 : 10 h light/dark photocycle at 25/20 °C, with a light intensity of 100–170 W·m^−2^ and 60% relative humidity.

At 5 weeks, the plants were treated with different concentrations of cadmium chloride (50, 100 and 200 μm) and 1 mg samples (roots, stems, leaves) from the same plants were collected at time intervals of 0 h, 6 h, 12 h, 24 h, 3 days and 5 days. At each treatment, samples from three different plants were collected for replicates. All of the samples were quickly frozen in liquid nitrogen for total RNA preparation or stored at −70 °C until use.

### Sequence and structure analysis of ramie metallothionein‐like protein

DNA sequence analysis and comparison were performed using lasergene (https://www.dnastar.com) and blast (http://www.ncbi.nlm.nih.gov/) and the open reading frames of the sequences were identified using orf‐finder (http://www.ncbi.nlm.nih.gov/gorf/gorf.html). Amino acid sequences alignment and phylogenetic analysis were performed using clustal w (www.phylogeny.fr) and mega 6.0 respectively. Predictions of functional motif were performed via the Expasy proteomics server (http://www.expasy.org).

### Quantitative RT‐PCR (qRT‐PCR) analysis of BnMTL expression under Cd^2+^ stress

Tissue samples were collected and saved in a liquid nitrogen container. Until all of the samples from different treatments and time points (0 h, 6 h, 12 h, 24 h, 3 days and 5 days) were collected, the total RNA were extracted using a Trizol kit (Invitrogen, Carlsbad, CA, USA) and quantified using a NanoDrop (Gene Co., Beijing, China) for the independent qRT‐PCR analysis. The first‐strand cDNA synthesis was performed with 1 μg of total RNA using the Marathon™ cDNA Amplification Kit (Clontech, Palo Alto, CA, USA) in accordance with the manufacturer's recommendations and the qRT‐PCR analysis was performed using gene‐specific primers and SYBR Green (Invitrogen) dye detection on a CFX96 system (Bio‐Rad, Hercules, CA, USA). The specific primers were designed using oligo 5 (https://www.oligo.net) and the 18s rRNA gene was used as a reference gene. The primers used to amplify 18s rRNA and BnMTL were: 18s rRNA, forward: TGACGGAGAATTAGGGTTCGA; 18s rRNA, reverse: CCGTGTCAGGATTGGGTAATTT; BnMTL, forward: ATGGGTTGCCCTTGTGGAAAC; BnMTL, reverse: TTGATTGCAAGAGCAGCTTGAG.

### Expression and western blotting analyses of BnMTL

Using the specific primers BnMTLF (5′‐GGAATTCATGGGTTGCCCTTGTGGAAAC‐3′) and BnMTLR (5′‐CAAGCTTTTGATTGCAAGAGCAGCTTGAG‐3′), the ORF fragment encoding the mature peptide (MGCPCGNNCQCGSSCACGGNSHTATEPSGCNCGPNCSCGSSCSCNQ) was obtained. It was purified using agarose gel electrophoresis, digested with *Eco*RI and *Hind*III enzymes and ligated into the *Eco*RI‐/*Hind*III‐digested expression vector pET‐30a (Novagen, Madison, WI, USA). The constructed plasmid was transformed into competent BL21 (DE3) cells for expression of the TRX (thioredoxin)‐6His‐BnMTL fusion protein and induced with 1 mm isopropyl‐d‐thiogalactoside (IPTG) for 6 h at 30 °C. The bacterial pellets were harvested by centrifugation and lysed by the lysis solution (50 mm Tris–HCl, pH 8.0, 50 mm NaCl, 0.5% Triton X‐100, 2 mg·mL^−1^ lysozyme). After sonication, the supernatants were recovered by centrifugation and subjected to Ni^2+^‐NTA column chromatography for purification of the recombinant fusion protein. The purified recombinant fusion protein was dialyzed and dissolved in PBS (pH 7.4) to a final concentration of 1 mg·mL^−1^. The fractions containing BnMTL were collected, concentrated with poly(ethylene glycol) 2000, dialyzed in double‐distilled water for desalination and finally lyophilized 22. Analysis of the purified recombinant BnMTL was carried out using Tricine‐SDS/PAGE.

Western blot analysis of target protein was performed according to the standard protocol. Briefly, the recombinant protein was separated on a 12% SDS/PAGE gel, which was semi‐dry transferred at 15 V for 30 min to 0.45 mm poly(vinylidene difluoride) membrane (Bio‐Rad), immunoblotted with anti‐His Tag mouse monoclonal antibody (dilution 1 : 5000; BOSTER, Wuhan, China). Next, the IgG goat anti‐mouse antibody conjugated with horseradish peroxidase was used as a secondary antibody (dilution 1 : 5000) and a diaminobenzidine kit was used for the visualization of the protein band.

### Assay of the Cd^2+^ tolerance and accumulation in pET30‐BnMTL/BL21

The cells of *E. coli* strain BL21 (DE3) that transformed with pET30a‐BnMTL and pET30a (control) were cultured in Luria–Bertani medium, and then the cell concentration was using the *D*_600_ measurements. When *D*_600_ of the bacterium liquid reached 0.2, the transformed *E. coli* cells containing pET30a‐BnMTL cells were induced with 1 mm IPTG in a 100 mL flask and simultaneously treated with different types of Cd^2+^. The *D*_600_ values were measured every 1 h to determine the growth rate and tolerance of cells in Cd^2+^ treatment.

To assess the Cd^2+^ binding capacity of the BnMTL, the flasks were supplemented with CdCl_2_ at concentration of 0.2 mm because the recombinant cells can growth normally in this concentration. Following the induction and Cd^2+^ treating, the accumulated Cd^2+^ (g^−1^ by dry weight) in *E. coli* cells was measured in accordance with the method described by Pan *et al*. 23: Cell samples (0.05 g) were placed into 50 mL porcelain crucibles and heated in a muffle furnace at 500 ± 25 °C for 8 h and then 10 mL of mixed acid (HNO_3_:HClO_4_ = 3 : 4) was added to each crucible. The porcelain crucibles were cooled at room temperature and then heated again under gentle heat until no carbon residues were visible. Subsequently, 10 mL of 8.3% HCl was added to dissolve remaining residues. The liquid solutions were then analyzed by flame atomic absorption spectrometry. A Cd^2+^ hollow cathode lamp was used as light source operated at 3.5 mA. The wavelength was set at 228.8 nm resonance line, the spectral bandpass at 0.5 nm and the measurements carried out in an air/acetylene flame.

The Cd^2+^ binding ability assay was performed in triplicate and differences between the treatments were examined for statistical significance using Duncan's test (*P* < 0.05, ANOVA). HCl solution was used as blank. The limit of detection was calculated as the analyte concentration equal to three times the SD of the blank signal divided by the slope of the calibration curve.

### Nucleotide sequence accession number

The nucleotide sequence data of the ramie metallothionein‐like gene (*BnMTL*) have been submitted to the nucleotide sequence databases (GenBank) under accession number MH481283.

## Results and Discussion

**Figure 1:**
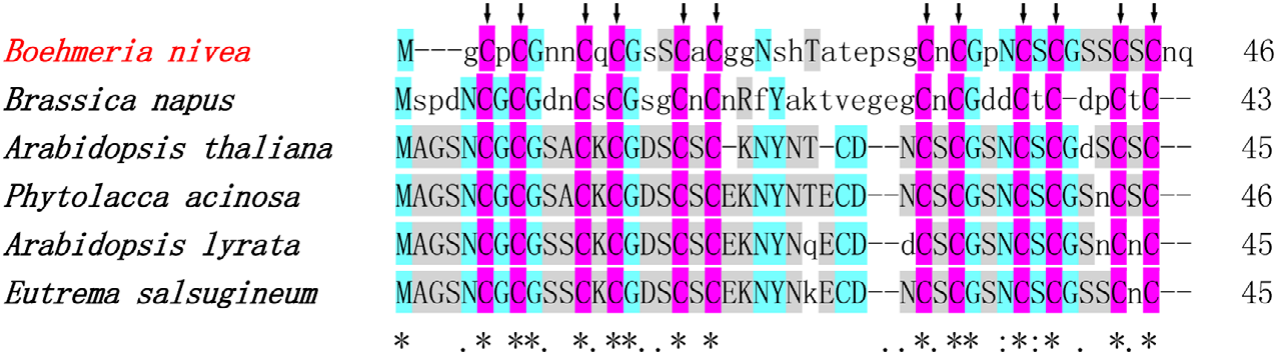
Amino acid sequence alignment of metallothionein‐like protein for *Boehmeria nivea* with other plants: *Brassica napus* (ADP37975), *Arabidopsis thaliana* (NP_172240), *Phytolacca acinosa* (AEP14524), *Arabidopsis lyrata* (XP_020870902) and *Eutrema salsugineum* (XP_006417787). Identical amino acid residues are indicated in blue and purple. The arrows indicate the conserved cysteine residues.

Expression profiling of Cd^2+^ response genes in root of ramie has been reported previously, with 36 unigenes from the cysteine and methionine metabolic pathway being up‐regulated 11. In the present study, the *BnMTL* gene cloned from ramie encodes a putative 46 amino acid protein with a molecular mass of 4.38 kDa. The ramie *BnMTL* is a typical micro‐molecular metallothionein‐like protein with a low molecular weight 1, 2, 24, 25, with its cysteine residues organized in two rich domains. Phylogenetic analysis suggested the *BnMTL* gene to be a type I metallothionein protein as a result of the equal distribution of six C‐X‐C motifs on both the N‐ and C‐terminal ends of the protein separated by a Cys‐poor linker. It is interesting to note that the length of the Cys‐poor linker region in *BnMTL* varies among different plant species (Fig. 1). These structural characteristics suggested the possible involvement of *BnMTL* in heavy metal detoxification and also that these residues may serve as primary chelating sites.

**Figure 2:**
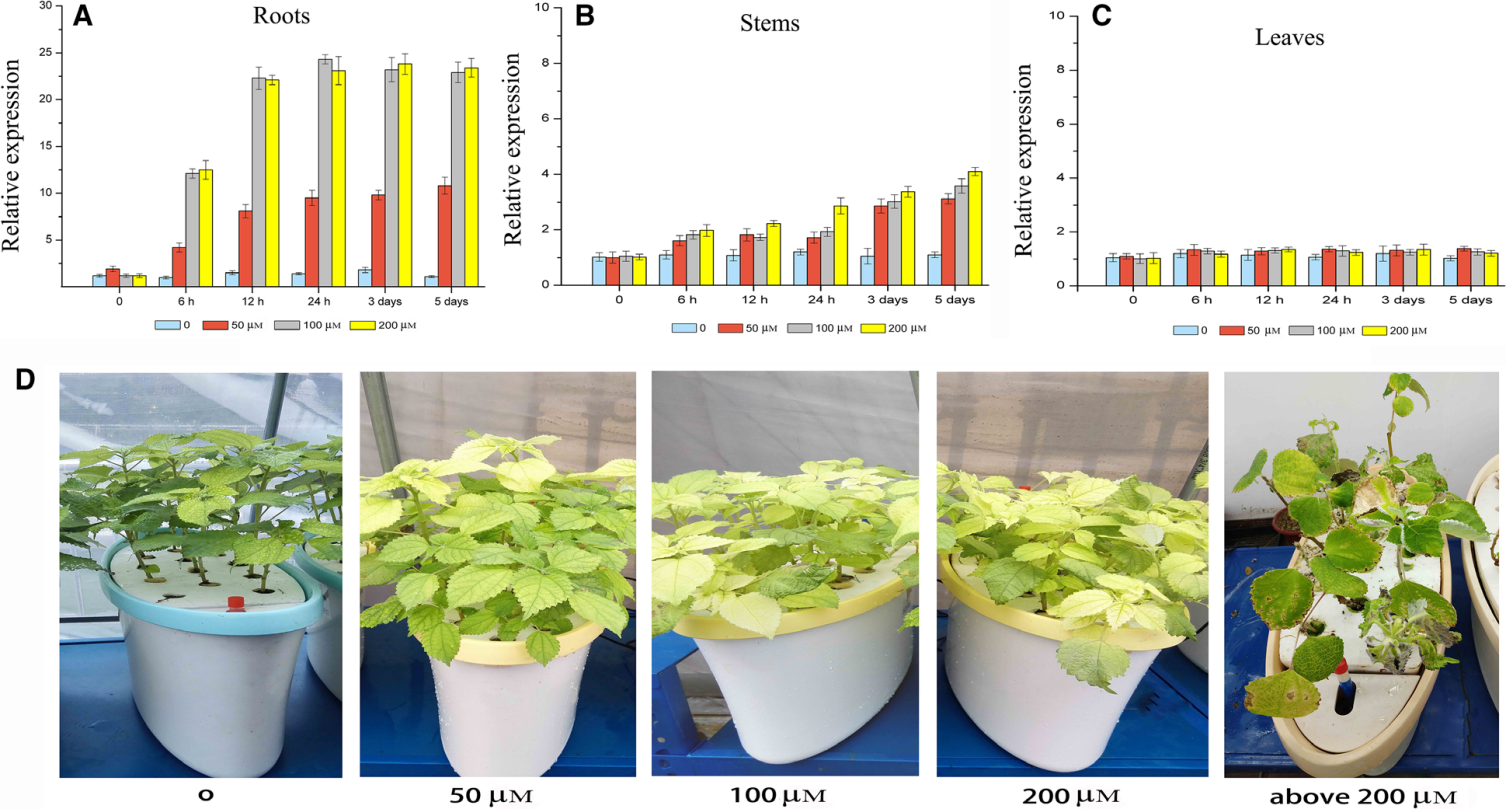
The expression profiles of BnMTL and the growth status of hydroponic ramie plant treated with various levels of Cd^2+^ stress. BnMTL expression in the roots (A), steams (B) and leaves (C) of hydroponic ramie cultured in the presence of different concentration of Cd^2+^ for the indicated periods were measured by qRT‐PCR. (D) Growth status of ramie plant treated with various levels of Cd^2+^ after 2 weeks. The value represents the mean ± SD of three biological replicate and three technical replicates were conducted for each organ.

The *BnMTL* genes were dramatically up‐regulated in ramie roots when exposed to various concentrations of cadmium chloride (Fig. 2). Similar results were found in several different plants such as *Avicennia germinnas* 26 and *Arachis hypogaea* 27. Studies on expression patterns of metallothionein in tomato demonstrated the best expression at an approximately 50 μm Cd^2+^ concentration 28. The results obtained in the present study, however, indicated significant up‐regulation of the gene at doses of 100–200 μm Cd^2+^ mainly induced in the roots (Fig. 2). On the other hand, a high Cd^2+^ concentration above 200 μm revealed an extremely harmful effect (Fig. 2) to ramie plants, which is in consistent with the studies conducted in tomato by Tombuloglu *et al*. 29, who reported the expression of tomato metallothionein gene to be decreased at higher Cd^2+^ doses, although the same studies also reported decreased expression at even lower doses. The different responses may be associated with physiological functions in ramie such as the hormonal status of tissues, tissue type and heavy metal uptake. The regulation of gene expression represents the first level of integration between environmental stress and the genome 30. Ramie is a perennial herb plant with developed underground roots, a high accumulation of heavy metal Cd^2+^ and BnMTL expression in roots rather than stems or leaves, consistent with the fact that the roots are the main organ for this species to adapt to a stress environment 31. Comprehensive consideration of the growth status, biomass accretion and total Cd content (Table 1) shows that these will provide an advantage for using ramie as a bast fiber or in some other multi‐purpose use, including as a candidate plant for phytoremediation of Cd polluted soil.

**Table 1:** The total Cd content and growth status of ramie treated with different concentrations of Cd^2+^.

Cd^2+^ concentration (μm)	Cd content			Growth status		
	Roots (mg·kg^−1^)	Stems (mg·kg^−1^)	Leaves (mg·kg^−1^)	Plant height (cm)	Stem width (mm)	Dry weight (g)
0	NA	NA	NA	30.15 ± 1.50	5.170 ± 0.200	9.30 ± 0.25
50	2560 ± 115	115 ± 8	6.08 ± 0.95	29.63 ± 1.15	4.985 ± 0.135	8.90 ± 0.20
100	3550 ± 150	183 ± 15	8.25 ± 1.21	25.15 ± 1.20	4.555 ± 0.121	5.80 ± 0.12
200	3490 ± 163	190 ± 11	8.63 ± 1.00	23.75 ± 1.23	4.330 ± 0.129	5.55 ± 0.10

The value are shown as the mean ± SD. NA, not available.

**Figure 3:**
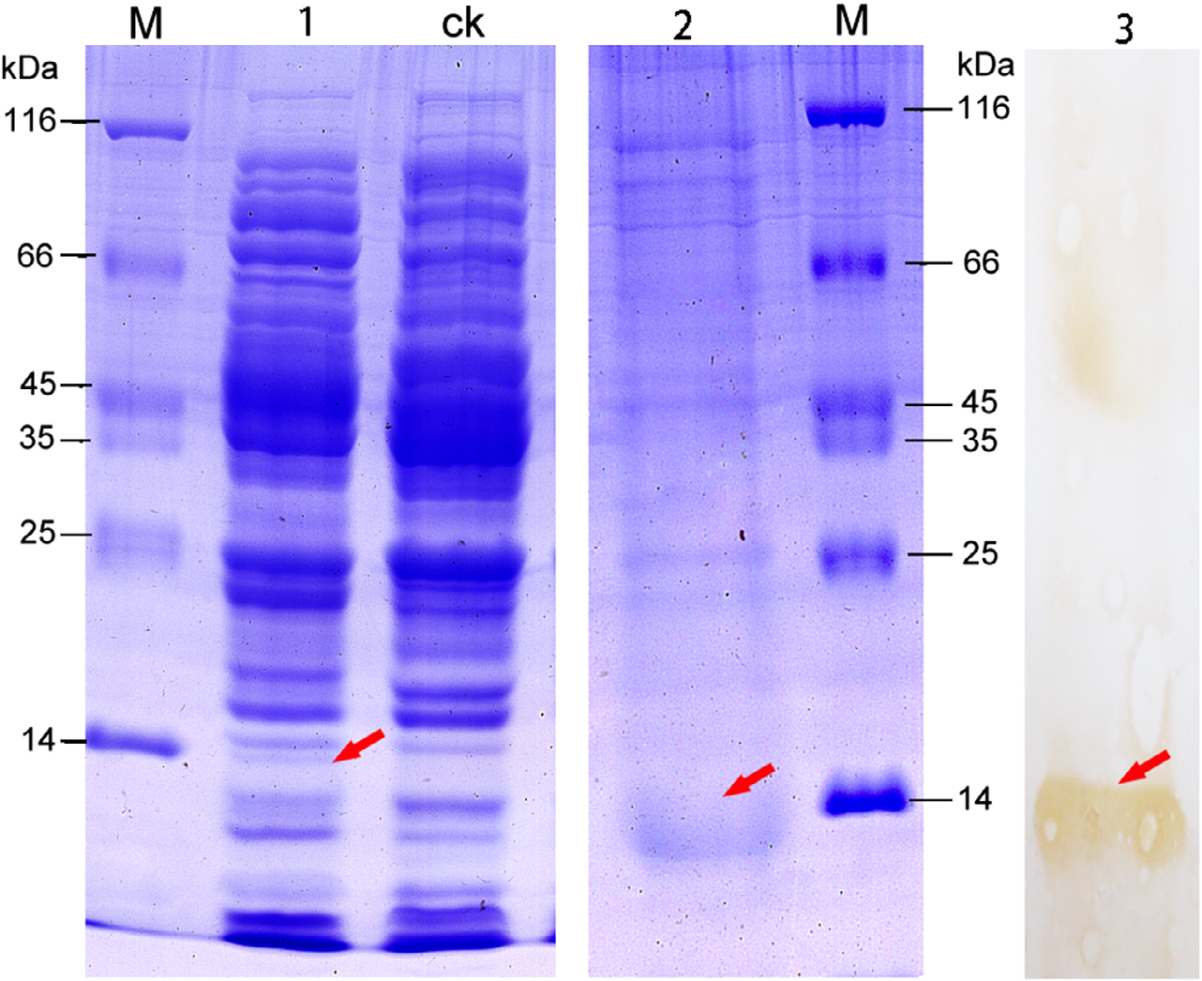
SDS/PAGE and western blot analysis of recombinant BnMTL. Lanes: M, standard protein molecular mass markers; 1, BnMTL from supernatant of *E. coli* BL21 cell lysates; 2, purified BnMTL; 3, western blotting of BnMTL from supernatant of *E. coli* BL21 cell lysates; The target bands of BnMTL are indicated by the arrows.

In an effort to characterize the Cd^2+^ binding properties, the *BnMTL* was heterologously overexpressed in *E. coli* cells. Analysis of protein expression using Tricine‐SDS/PAGE showed purified homogenous recombinant *BnMTL* (fusion protein included the tags from the pET30a vector) with a molecular mass of approximately 14 kDa (Fig. 3, lane 1). The low molecular weight of metallothionein protein and its susceptibility to proteolysis were ascribed to be the cause of difficulty in native metallothionein protein isolation in plants, in addition to the difficulties with respect to its purification as a result of instability in the presence of oxygen 20, 32, 33. The expression of *BnMTL* was further confirmed by western blotting analysis using His‐tag antibodies (Fig. 3, lane 3).

**Figure 4:**
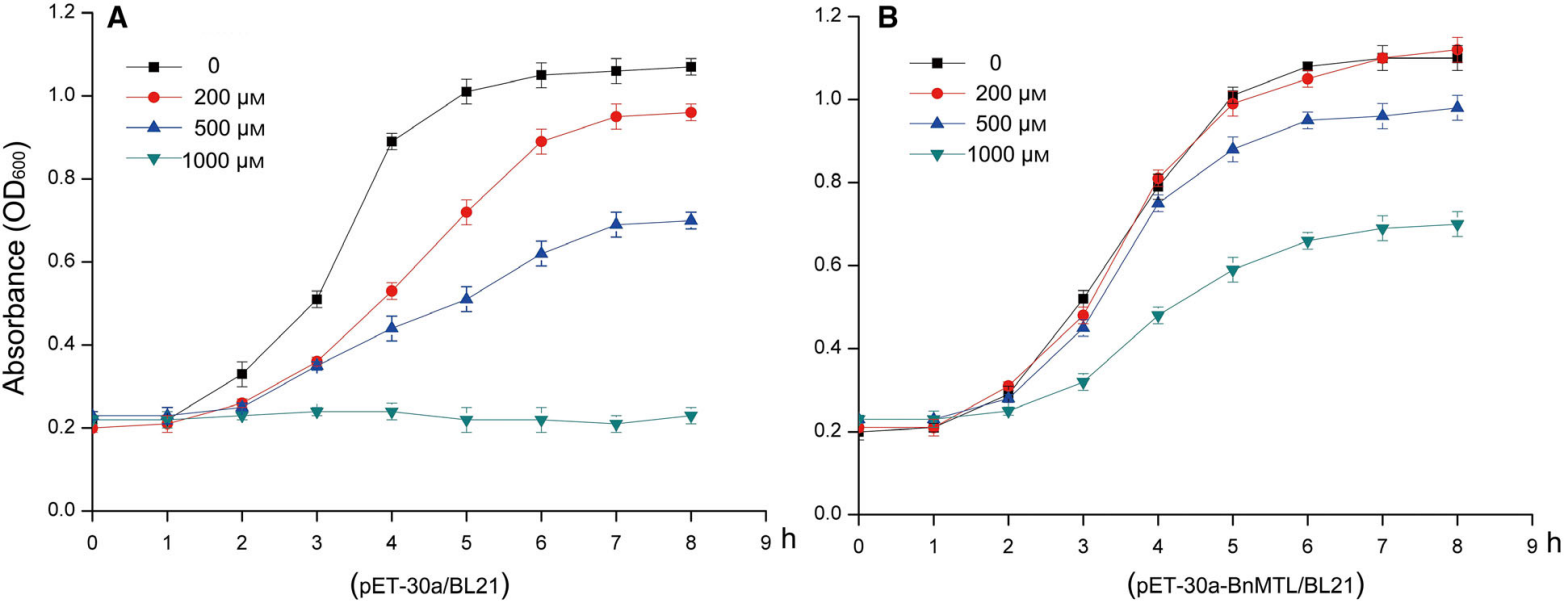
Cd^2+^ tolerance analysis of recombinant *E. coli* cells expressing BnMTL. The growth status of pET30a‐BnMTL/BL21 (A) and pET30a /BL21(B) in different Cd^2+^ concentrations. The cell concentration was calculated from the *D*_600_ measurements. Data represent the means of three experiments and error bars represent the SD.

The recombinant cells grown in the Luria–Bertani medium supplemented with different concentrations of cadmium chloride (0, 200, 500 and 1000 μm) showed no significant difference with respect to the growth rate of pET30a‐BnMTL/BL21 recombinant cells cultured under 0 and 200 μm Cd^2+^. After 6 h of culture, they reached the stationary phase with a OD_600_ value of 1.1. The growth rate of recombinant cells was inhibited at a higher concentration of cadmium ions (500 and 1000 μm), with the cells attaining a stationary phase under the 1000 μm Cd^2+^ ions stress when the OD_600_ value was only 0.6. The growth of control cells (pET‐30a/BL21) was also found to be significantly inhibited in the Cd^2+^ concentration of 200 and 500 μm (Fig. 4).

**Figure 5:**
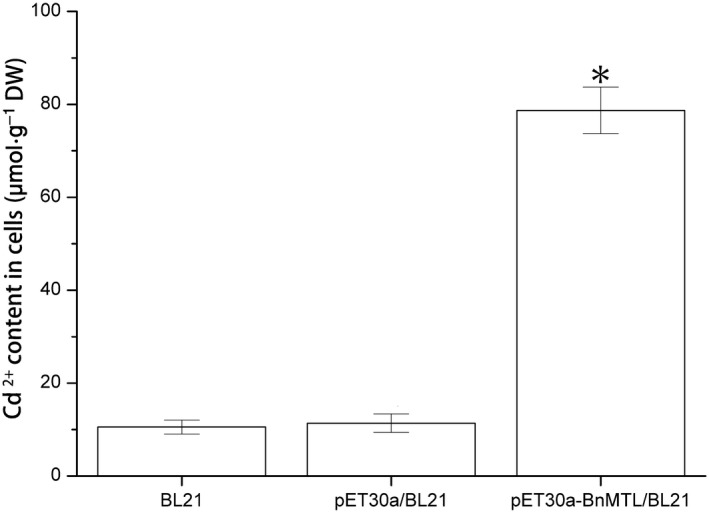
The accumulation of Cd^2+^ ions in the pET30a‐BnMTL/BL21 and the control *E. coli* cells (BL21 and pET30a/BL21). The value represents the mean ± SD of three biological replicates. Statistical significance was based on Duncan's test. *Significant difference between *E. coli* cell samples at *P* < 0.05.

The accumulation of Cd^2+^ (μmol·g^−1^ dry weight) in *E. coli* cells was also investigated. The highest levels of Cd^2+^ were detected in *E. coli* cells expressing *BnMTL* (82.16 μmol·g^−1^ dry weight). The recombinant *E. coli* cells expressing *BnMTL* exhibited the highest accumulation of Cd^2+^ ions, with significantly higher levels compared to those of controls (pET30a/BL21 and BL21 strains), being almost eight‐fold greater than the controls (Fig. 5). The expression patterns of the recombinant BnMTL suggested that the cells transformed with the recombinant plasmids pET30a‐BnMTL had a high tolerance to Cd^2+^ stress and can be grown well in the concentration of Cd^2+^ under 1000 μm. This is in accordance with an enhanced tolerance to Cd^2+^ in recombinant strains expressing metallothionein being demonstrated in *Musca domestica* 34, biofuel plant *Jatropha curcas* 35, *Anabaena* sp. 36 and bacterial metagenome 37. There was a direct relationship between increased metallothionein gene expression and survival of the recombinant *E. coli* cells. Furthermore, a high level of cadmium ions accumulated in the recombinant *E. coli* cells harboring pET30a‐BnMTL, indicating that expression of *BnMTL* could enhance tolerance in cells to the Cd^2+^ ion concentration and promote the accumulation of Cd^2+^. The results obtained in the present study may help to confront to Cd^2+^ pollution using the overexpression of the metallothionein gene in recombinant bacteria.

## Conclusions

In the present study, we have cloned and identified a low weight metallothionein‐like protein gene (*BnMTL*) from the potential phytoremediation plant ramie. Tissue‐specific expression analysis showed the expression of *BnMTL* to be regulated by Cd^2+^ treatment and induced in roots. As a result of difficulty in isolating native metallothionein protein because of its low molecular weight and susceptibility to proteolysis, we heterologously overexpressed BnMTL in *E. coli* cells. The Cd^2+^ tolerance and accumulation analysis demonstrated that BnMTL improved the Cd^2+^ tolerance of the recombinant *E. coli* cells. Such work lays a foundation for defining the roles of BnMTL in Cd chelation and detoxification.

## Conflict of interest

The authors declare no conflict of interest.

## Author contributions

GG and ZA conceived the study and, together with YC, supervised its conduct. CJ and CK performed the gene cloning experiment and qRT‐PCR analysis. CP, GG and ASA performed the heterologously overexpress of BnMTL and the cadmium‐binding activity assay. All authors analyzed and discussed the data and contributed to writing the manuscript.
